# Newborn care in Indonesia, Lao People’s Democratic Republic and the Philippines: a comprehensive needs assessment

**DOI:** 10.1186/1471-2431-14-46

**Published:** 2014-02-15

**Authors:** Els Duysburgh, Birgit Kerstens, Melissa Diaz, Vini Fardhdiani, Katherine Ann V Reyes, Khamphong Phommachanh, Marleen Temmerman, Basil Rodriques, Nabila Zaka

**Affiliations:** 1 International Centre for Reproductive Health (ICRH), Ghent University, De Pintelaan 185 UZP114, 9000 Ghent, Belgium; 2 HERA, Right to Health & Development, Laarstraat 43, 2840 Reet, Belgium; 3 Jl. Meditran X Blok.M30/7 Pondok Ranji, Ciputat Timur, Tangerang Selatan, Banten 15412, Indonesia; 4 Unit 11-O Torre Venezia Suites, 170 Timog Avenue cor Scout Santiago Street, Quezon City 1103, Philippines; 5 Ban Nongthathai, Chanhthabuly District, Vientiane, Laos; 6 Department of Reproductive Health and Research, World Health Organisation, Avenue Appia 20, 1211 Geneva, Switzerland; 7 UNICEF East Asia Pacific Regional Office, 19 Phra Atit Road, Chanasongkram, Phra Nakorn, Bangkok 10200, Thailand

**Keywords:** Newborn care, Needs assessment, Quality of care, Equity, Healthcare policy, South-East Asia

## Abstract

**Background:**

Between 1990 and 2011, global neonatal mortality decline was slower than that of under-five mortality. As a result, the proportion of under-five deaths due to neonatal mortality increased. This increase is primarily a consequence of decreasing post-neonatal and child under-five mortality as a result of the typical focus of child survival programmes of the past two decades on diseases affecting children over four weeks of age. Newborns are lagging behind in improved child health outcomes. The aim of this study was to conduct a comprehensive, equity-focussed newborn care assessment and to explore options to improve newborn survival in Indonesia, Lao People’s Democratic Republic (PDR) and the Philippines.

**Methods:**

We assessed newborn health policies, services and care in the three countries through document review, interviews and health facility visits. Findings were triangulated to describe newborns’ health status, the health policy and the health system context for newborn care and the equity situation regarding newborn survival.

**Results:**

Main findings: (1) In the three countries, decline of neonatal mortality is lagging behind compared to that of under-five mortality. (2) Comprehensive newborn policies in line with international standards exist, although implementation remains poor. An important factor hampering implementation is decentralisation of the health sector, which created confusion regarding roles and responsibilities. Management capacity and skills at decentralised level were often found to be limited. (3) Quality of newborn care provided at primary healthcare and referral level is generally substandard. Limited knowledge and skills among providers of newborn care are contributing to poor quality of care. (4) Socio-economic and geographic inequities in newborn care are considerable.

**Conclusions:**

Similar important challenges for newborn care have been identified in Indonesia, Lao PDR and the Philippines. There is an urgent need to address weak leadership and governance regarding newborn care, quality of newborn care provided and inequities in newborn care. Child survival programmes focussed on children over four weeks of age have shown to have positive outcomes. Similar efforts as those used in these programmes should be considered in newborn care.

## Background

Worldwide neonatal mortality (deaths during the first 28 days of life) has declined 32% from 32 deaths per 1,000 live births in 1990 to 22 per 1,000 live births in 2011 [1]. However, this decline is slower than that of under-five mortality for the same period. Between 1990 and 2011, global under-five mortality dropped on average 2.5% per year while neonatal mortality declined 1.8% annually [1]. As a result, worldwide the proportion of under-five deaths due to neonatal mortality increased, from 36% (4.362 million) in 1990 to 43% (2.955 million) in 2011 [1]. This increase is primarily a consequence of decreasing post-neonatal (deaths after 29 days of life and before age one) and child mortality (deaths between age one and age five) in children under five from infectious diseases such as measles, pneumonia, diarrhoea, malaria, and AIDS [2,3]. Child survival programmes have typically focused on these diseases affecting children over four weeks of age, resulting in a larger reduction in mortality in this group of children compared to the newborn group [3,4]. These findings urge the need for focus on newborn health, as newborns are those lagging behind in improved child health outcomes.

The leading causes of neonatal death globally are complications of preterm birth, intra-partum related causes and sepsis or meningitis [2,4]. The majority of these neonatal deaths can be prevented with known, low-tech and effective interventions such as improved hygiene at birth, exclusive breastfeeding, newborn resuscitation, management of newborn infections and simple approaches to keeping babies warm [5,6].

We hereby present the results of the newborn needs assessment we conducted in Indonesia, Lao People’s Democratic Republic (PDR) and the Philippines. Its objective was to conduct a comprehensive, equity-focussed assessment and to explore options to improve newborn survival. An independent team of researchers conducted the study on behalf of UNICEF East Asia and Pacific Regional Office.

## Methods

### Study setting

This study took place in Indonesia, Lao PDR and the Philippines, three countries located in South-East Asia.

Countries located in the UNICEF East Asia and Pacific region, having a high neonatal mortality proportion among their under-five deaths and, having not yet achieved Millennium Development Goal 4 (aiming to reduce the under-five mortality rate by two-thirds between 1990 and 2015) were defined as being eligible to participate in this study. Participation of eligible countries was discussed with respective government counterparts and Indonesia, Lao PDR and the Philippines accepted to take part in this study. Some newborn health-related indicators of these three countries are given in Table 1.

**Table 1:** Newborn health-related indicators, Indonesia (2012), Lao PDR (2011-12) and the Philippines (2008 and 2011)

***Newborn service***	***Coverage of service (%)***		
	***Indonesia***	***Lao PDR***	***Philippines***
Skilled birth attendance*	83	42	72
Hep B (first dose)	85	37 (given at birth)	87
Hep B (fourth dose)	41	56	67 (third dose)
BCG vaccination	89	78	92
Postpartum care for newborn within two days	48	47	not available
Newborns that have a reported birth weight (based on either a written record or the mother’s recall)	89	43	73
Newborns that received breastfeeding within 1 hour of birth	49	39	54

*Skilled birth attendant includes doctor, nurse, midwife, and auxiliary nurse/midwife.

Sources [7-10].

### Study set-up

We assessed newborn health policies, services and care and the equity of these services and care in the three countries through document review, semi-structured interviews and health facility visits.

The document review included national policy documents, newborn health guidelines, routinely collected data, reports on newborn health status and scientific papers published in peer reviewed journals. Documents related to maternal and newborn care were collected using library and web-based search engines. We used PubMed to search for peer reviewed papers, and Google and Google Scholar to find grey literature. Documents and papers found as such were supplemented with documents provided by the UNICEF advisors and programme staff at the regional and country offices.

In each country we conducted interviews with key informants including policy makers at national, provincial and district level, representatives of health professional organisations, United Nations organisations, bilateral and multilateral development agencies, non-government organisations and civil society organisations, healthcare providers in the public and private sector, and mothers. In Indonesia, four policy makers in newborn health and managers belonging to government institutions, and 26 belonging to other organizations, were interviewed. In PDR these were seven belonging to government institutions and 14 to other organizations and in the Philippines eight and ten respectively. In each of the visited health facilities we conducted several interviews with providers (doctors, midwives, nurses and community health workers/village midwives attached to the health facility) and mothers. These were brief interviews focussing on the providers’ work with newborns and their comments and perception regarding the situation and care of the newborns. The interviews with mothers focussed on their perception and satisfaction with the provided newborn care. An interview guide was developed and used during the interviews. The interviewers took notes of the answers given.

Per country, between four and eight health facilities, including primary healthcare and referral facilities, were visited in both rural and urban areas. Only facilities providing antenatal, childbirth and postpartum services for mother and newborn and corresponding national norms regarding medical infrastructure, equipment and staffing, were eligible to be selected for these visits. The regions and facilities were selected by the researchers with inputs from the national UNICEF staff and the respective ministries of health. The main objective of these visits was to observe the conditions for newborn care and its implementation and to talk with healthcare providers and clients about newborn care. As standard for good newborn care and services we used the available national newborn policies and guidelines which are based on internationally recommended policies and guidelines. At each facility we checked if commodities and drugs needed for newborn care were available, and if providers knew the kind of routine newborn care they have to provide, how to manage and treat complications occurring in newborns and how to conduct emergency newborn care.

### Data collection and analysis

The document review was conducted in November 2012, the interviews and health facility visits took place in November and December 2012. The document review, interviews and health facility visits were conducted by an independent team of researchers consisting of five medical doctors with public health and MNCH background and one health economist. Three of the researchers were European, three came from the respective Asian countries included in this study.

Quantitative data found by document review and review of available data were organised in tables and interpreted in the frame of the newborn care assessment. The qualitative findings from the document review, interviews and health facility visits were triangulated to describe newborns’ health status, the health policy and the health system context for newborn care, and the equity situation regarding newborn care and services. The analysis was based on the ‘six building blocks’ of a health system as defined by the World Health Organisation: (1) service delivery, (2) human resources, (3) essential medicine and technologies, (4) health financing, (5) health information systems, and (6) governance and leadership [11].

### Ethics

UNICEF East Asia and Pacific region representatives discussed participation of eligible countries with the respective government counterparts and only those that accepted to participate in the assessment were included. None of these countries required ethics approval from the government for this study. Most likely because this assessment was not categorised as primary research as the research was based on readily available data and documents. Oral informed consent was obtained from all key informants before enrolment in the study.

## Results

During the previous two decades, insufficient progress has been made in reducing neonatal mortality in Indonesia, Lao PDR and the Philippines (see Table 2). The neonatal mortality rate (NMR), defined as numbers of deaths in the first 28 days of life per 1,000 live births, is at present 32 deaths per 1,000 live births in Lao PDR, 19 deaths per 1,000 live births in Indonesia and 16 deaths per 1,000 live births in the Philippines [7-9]. Neonatal mortality has slowly been decreasing in the three countries since 1990 but, in line with what is observed globally and due to the same reasons, namely greater focus on and better results in improving health of children older than one month, this decline is lagging behind the decline of under-five mortality. There is still insufficient insight in the causes of mortality, as neonatal and perinatal death audits are not or not regularly enough conducted. In Lao PDR and in the Philippines a system for perinatal and neonatal death audits does not exist. In Indonesia a system exists, however it is poorly implemented and audits are not performed systematically.

**Table 2:** Trends in neonatal and under-5 mortality for five year periods, 1990-2012

	***5 year periods***					***Percentages decrease between 1990***-***1994 and most recent mortality rate***
	***1990***-***1994***	***1995***-***1999***	***2000***-***2004***	***2005***-***2009***	***2010***-***2012***	
**Indonesia**						
Neonatal mortality rate^*^	30	22	20	19	19	37%
Under-5 mortality rate^**^	81	58	46	44	40	51%
**Lao PDR**						
Neonatal mortality rate	54	49	52	42	32	41%
Under-5 mortality rate	170	150	146	106	79	54%
**Philippines**						
Neonatal mortality rate	18	18	17	16	-	11%
Under-5 mortality rate	54	48	40	34	-	37%
**World**						
Neonatal mortality rate	32	-	-	-	22	31%
Under-5 mortality rate	87	82	73	63	53	39%

Sources [1,7-9,12-18].

^*^numbers of deaths in the first 28 days of life per 1,000 live births.

^**^numbers of deaths before reaching the age of five per 1,000 live births.

Despite the still relatively high neonatal mortality, national comprehensive newborn policies in line with international standards exist in the three countries. Table 3 gives an overview of the documents identified and reviewed as part of this assessment. Policies, strategies, guidelines and legislation published during the last two decennia directly related to and relevant for maternal, newborn and child health (MNCH) were included. Most policy makers and health managers interviewed mentioned that the implementation of these policies and guidelines remains poor. Providers often mentioned that they were not aware of the availability of these guidelines and do not know what kind of newborn care they are supposed to provide. Key informants often stated the decentralisation of authorities and relegation of responsibilities to provincial, district and municipality levels as an important factor hampering the implementation. They mentioned that decentralisation created confusion regarding the roles and responsibilities for newborn care in health management, including in financing, planning and implementation. Interviews and reviewed reports also made it clear that management capacity and skills at provincial, district and municipality level were often limited and are as such jeopardising the implementation of good quality newborn care. Nevertheless, lots of key informants were positive regarding decentralisation, mentioning that decentralised health systems bear lots of opportunities such as the possibility to tailor healthcare to the local context and needs. Decentralisation of the health system was initiated in the Philippines in 1991, in Indonesia in 2001 and in Lao PDR in 2005.

**Table 3:** Policies, strategies, guidelines and legislations related to maternal, newborn and child health in Indonesia, Lao PDR and The Philippines (in chronological order)

***Year***	***Policies, strategies, guidelines and legislations***
**Indonesia**	
***Relevant for maternal***, ***newborn and child health***	
2010-2014	Health Strategic Plan 2010 – 2014 – MoH (HEALTH MINISTER DECREE NO. 021 / MENKES / SK / I / 2011)
2005-2025	National Long‒Term Development Plan (RPJPN 2005‒2025) (Based on Law No. 17/2007)
***Related to maternal***, ***newborn and child health***	
2011	Guidelines for management of newborn asphyxia for midwives, Directorate General of Nutrition and Maternal and Child Health, Ministry of Health, 2011
2011	Guidelines for management of low birth weight (LBW) for midwives and nurses, Directorate General of Nutrition and Maternal and Child Health, MoH, 2011
2010	Essential Newborn Care, Technical Guideline To Basic Healthcare, pocket book, MoH Republic of Indonesia, 2010 (English and Bahasa version available)
2010	Guidelines for newborn care (PANDUAN PELAYANAN KESEHATAN - BAYI BARU LAHIR -BERBASIS PERLINDUNGAN ANAK), Child health unit, MoH, 2010
2001-2010	National 2001-2010 Making Pregnancy Safer Strategy
***MNCH related legislations***	
2012	Regulation on Exclusive Breastfeeding (PP Nomor 33 Tahun 2012 about “Pemberian Air Susu Ibu Eksklusif”). Published by the Government of Indonesia (GoI), signed by the president on 1 March 2012
2011	Permenkes No. 2562/Menkes/Per/XII/2011 -- > Technical Guidelines for Jampersal (MNC health insurance). Signed by Minister of Health, 27 December 2011
2008	Kepmenkes No. 603/Menkes/SK/VII/2008 -- > Implementation of Operational Guideline for Mother-Friendly-and-Baby-Friendly Hospital. Signed by Minister of Health, July 2008
**Lao PDR**	
***Relevant for maternal***, ***newborn and child health***	
	Health Strategy 2020
2011-2015	Seventh Five-Year Health Sector Development Plan 2011-2015
2009	National Policy on Human Resources for Health 2009
2008	National Nutrition Policy 2008
2006-2010	National Strategic Action Plan on HIV/AIDS/STI 2006-2010
2005	Lao Health Master Plan 2005
2003	National Intestinal Helminth Prevention and Control Policies 2003
2002	Hygiene, Prevention and Health Promotion Law 2002
2000	National Policy on HIV/AIDS control 2000
2000	Primary Health Care Policy 2000
1999	National Population and Development Policy 1999
***Related to maternal***, ***newborn and child health***	
2010	National Policy and Holistic Early Childhood development 2010
2009-2015	Strategy and planning framework for integrated package of maternal, neonatal and child health services 2009-2015
2008-2015	Skilled Birth Attendance Development Plan 2008-2015
2005	National Reproductive Health Policy 2005
2005	Regulation on the Promotion of Maternal and Child Health 2005
2004	Women Development and Protection Law 2004
1999	Prime Minister’s Decree-Establishment of the National Commission for Mother and Child 1999
	National Breastfeeding Policy
	Baby Friendly Hospital Initiative
**Philippines**	
2012	Responsible Parenthood and Reproductive Health Act *Republic Act No. 10354*
2011	Guidelines on the Certification of Health Facilities with Basic Emergency Obstetrics and Newborn Care Capacity *Administrative Order No. 2011*-*0014*
2010	The Aquino Health Agenda: Achieving Universal Health Care for All Filipinos *Administrative Order No. 2010*-*0036*
2010	Administration of Lifesaving Drugs and Medicines by Midwives to Rapidly Reduce Maternal and Newborn Mortality *Administrative Order No. 2010*-*0014*
2010	Revised Policy on Micronutrient Supplementation to support achievement of 2015 MDG Targets *Administrative Order No. 2010*-*0010*
2010	Policies and Guidelines for the Philippine National Blood Services and the Philippine Blood Services Network *Administrative Order No. 2010*-*0001*
2009	Adopting New Policies and Protocol on Essential Newborn Care *Administrative Order 2009*-*0025*
2009	Policies and guidelines on the Prevention of Mother to Child Transmission (PMTCT) of HIV *Administrative Order No. 2009*-*0016*
2009	Expanding the promotion of breastfeeding Act *Republic Act 10028*
2008	Implementing Health Reforms for the Rapid Reduction of Maternal and Neonatal Mortality *Administrative Order No. 2008*-*0029*
2007	Revitalization of the Mother-Baby Friendly Hospital Initiative in Health Facilities with Maternity and Newborn Care Services *Administrative Order No. 2007*-*0026*
2005	National Policy on Infant and Young Child Feeding Strategy *Administrative Order No. 2005*-*0014*
2004	Newborn Screening Act *Republic Act 9288*
2000	Early Childhood Care and Development Act *Republic Act 8980*
2000	Safe Motherhood Policy *Administrative Order 2000*-*0079*
1996	Code of Marketing of Breast milk Substitutes *Executive Order 51*
1992	Midwifery Act of 1992 *Republic Act 7392*
1992	The Rooming-In and Breastfeeding Act for hospitals and Health Facilities *Republic Act. 7600*

During interviews, many health policy makers and managers mentioned that in spite of the availability of health facilities providing good newborn care, the overall quality of newborn care was a major concern. They mentioned that substandard quality of care was a problem at primary healthcare and referral level. This was the case for routine newborn care, for case management of the sick newborn and for emergency newborn care. As important reasons for the poor quality they identified limited knowledge and skills for newborn care of health workers at all levels. These conclusions were confirmed by the findings of providers interviews, observations made during the health facility visits and available data on quality of care [7-9,19,20]. Many providers interviewed were unable to tell what kind of essential newborn care has to be provided. In some of the visited health facilities newborn resuscitation equipment was not available. Although routinely collected data on quality of newborn care was hard to be found, the available data shows room for improvement of newborn care quality (see Table 1, two last indicators). In this context, key informants at national, regional and district level often mentioned that supervision and mentoring of health staff, generally recognised as being important in delivering and maintaining good quality of care, was rather poor.

Data and results from reviewed reports and papers show that access to skilled health workers, mainly in rural and remote areas, remains limited, not only due to unequal distribution or lack of health staff and financial and geographic barriers but also because of local beliefs and practices [21-26]. This was also mentioned by policy makers, managers and health providers interviewed. We found the following data regarding the number of available skilled health workers: in Indonesia there are 19.9 healthcare professionals per 10,000 population (although this is believed to be an underreporting of the real situation as data from the private sector and from hospitals belonging to other ministries are not included) [27], in Lao PDR there are 8.2 healthcare professionals per 10,000 population [28] and in the Philippines 10.3 medical doctors, 15.5 midwifes and 40.0 registered nurses per 10,000 population [22]. In Indonesia and the Philippines there are sufficient midwives, nurses and medical doctors but most reviewed reports and consulted key informants stated that the unequal distribution of these health providers disadvantages the difficult to reach areas. Staff retention in these areas was identified to be challenging [21-26]. Apart from the unequal distribution and retention problems, overall workforce shortage is an additional problem in Lao PDR.

Based on findings from the document review and from interviews with key informants, we found that local practices, beliefs and myths, especially in Indonesia and Lao PDR, were influencing maternal and newborn health seeking behaviour [29-33]. Traditional birth attendants still have an important position in providing newborn care, especially in rural areas [32,34,35].

Geographic accessibility to newborn care is an issue especially for people living in remote and difficult to reach areas [35]. In Indonesia and the Philippines providing care at all islands is not easy to organise, while in Lao PDR reaching communities living in very remote areas without roads is challenging. Approaches to overcome the gaps in geographic accessibility are implemented, such as the deployment of village midwives in Indonesia and the establishment of village health stations and community health teams in the Philippines, although challenges in accessibility remain [21,36-39].

Strategies to reduce or eliminate financial barriers to newborn care exist in the three countries. In the Philippines and Indonesia respectively, premium- and tax-based national health insurance schemes covering newborn care for respectively the poor and for all citizens are in place [40-42]. In Lao PDR the government recently approved a policy for free delivery and care for children under five years of age [43]. Despite these strategies, financial barriers to newborn care remain. For example, the fact that transport costs are not covered by the insurance schemes in Indonesia and in the Philippines is a barrier to care for the poor and for those living in remote areas where transport costs can be high [44,45]. In Lao PDR an exact roll-out plan for the free of charge policy and the required budget were not yet available at the time of the country assessment.

The above findings on socio-cultural, geographic and financial access to newborn care are directly linked to the socio-economic and demographically observed inequities in newborn care. As Table 4 shows, neonatal mortality varies depending on socio-economic and geographic background. Mortality rates are highest among the most disadvantaged with higher rates found in the lowest wealth quintiles, among the less educated women and among rural residents [7-9].

**Table 4:** Neonatal mortality rate by socio-economic and geographic characteristics, Indonesia (2012), Lao PDR (2011/12) and the Philippines (2008)

***Characteristics***	***Neonatal mortality rate***		
	***Indonesia***	***Lao PDR***	***Philippines***
**Residence**			
*Urban*	15	22	13
*Rural*	24	39	20
**Mother**’**s education**			
*No education*	31	44	37
*Primary*	24	35	16
*Secondary* +	10	22	11
**Wealth quintile**			
*Lowest*	29	40	20
*Second*	21	47	19
*Middle*	23	43	15
*Fourth*	15	17	15
*Highest*	10	18	10
***Total NMR***	***19***	***36***	***16***

Source [7-9].

Coverage of newborn care shows the same inequities. For most care, such as early initiation of breastfeeding, newborns weighed immediately after birth and BCG vaccination, the coverage declines with lesser education and wealth level and is lower in rural areas compared to urban areas [7-9]. Lower coverage in the rural areas is also reinforced by the overrepresentation in the rural and more remote areas of a less educated and poorer population. The inequities in newborn care coverage are considerable despite the introduction of several initiatives and programmes such as the village midwifes initiative in Indonesia and insurance schemes introduced in Indonesia and the Philippines.

Finally, we would like to mention two important findings regarding health sector organisation jeopardising newborn care. Firstly, fragmentation of newborn care across several ministry of health departments in the three countries hampers prioritisation and efficient coordination and implementation of newborn care. And secondly, despite the importance of the private health sector in Indonesia and the Philippines, governmental regulation of and cooperation with this sector is weak. This may have a negative impact on newborn care and is also a missed opportunity to improve access to care.

## Discussion

Similar challenges for newborn care were identified in Indonesia, Lao PDR and the Philippines and show the need to improve access to quality newborn care. Opportunities identified to address this need include: (1) strengthening leadership and skills of health management, (2) improving quality of newborn care and (3) minimizing socio-economic and geographic inequities. Need for improvement of the quality of newborn care and for addressing the inequities in newborn care were also expressed in several recent studies [4,19,46].

Improved leadership and governance may enhance the implementation of newborn policies and improve the quality of care provided at the facilities. Clear responsibilities and roles of authority for all departments and all administrative levels therefore need to be defined. Additionally, management skills and capacity in planning, budgeting, and supervision at provincial, district and municipality level need to be improved [47].

Although evidence-based, cost-effective interventions for newborn care are known, the implementation of good quality newborn care remains a problem [5,48-52]. A precondition for health workers to provide good quality newborn care is that they receive high quality training. Guaranteeing quality pre- and in-service training in newborn care for all levels of health workers is crucial. This implies the existence of well-functioning accreditation, standardisation, regulation and monitoring systems of the training institutions which was identified in Indonesia as currently rather weak or missing [42]. In recent years, Lao PDR and the Philippines have invested a lot in improving pre-and in-service training on maternal, newborn and child health [53,54]. Despite this we found that, similar to other studies from the region, knowledge and skills to provide good quality newborn care were missing [20,55]. Special attention is needed to ensure that adequate skills training, including practice with patients, is part of the curricula. Another important although often neglected or poorly implemented tool to maintain and/or improve quality of care, is supportive supervision conducted at health facilities [4,54,56].

Having enough professional health workers equally distributed in the country is another requirement for providing good newborn care. While there is no gold standard for the sufficiency of the health workforce, WHO estimates that countries with fewer than 23 healthcare professionals (counting only physicians, nurses and midwives) per 10,000 population will be unlikely to achieve adequate coverage rates for the key primary healthcare interventions prioritized by the Millennium Development Goals [57]. This ratio is far from being reached in Lao PDR. In Indonesia and the Philippines this ratio seems to be easily reached, but the unequal distribution of staff in favour of the urban areas and retention problems, especially in rural areas, leads to staff shortages in some country regions. Employment of local health staff, task shifting and involvement of community health workers are strategies which might have a positive impact on this unequal distribution of health workers and on newborn health outcomes [56,58,59].

Several strategies to reduce socio-economic and geographic inequities in newborn health, are known and have proven to be successful [56,60-62]. In Indonesia, Lao PDR \and the Philippines some of these were introduced, such as: free newborn care in Lao PDR, introduction of health insurance schemes in Indonesia and the Philippines, and introduction of village midwifes in Indonesia and village health stations in the Philippines. Apart from the free newborn care in Lao PDR, all the other strategies were implemented more than one decade ago. Despite the long-lasting implementation of these strategies, the inequity in newborn mortality remains high as can be seen in Table 4. More focus on context specific approaches is needed [4,24,46]. A decentralised health system offers the opportunity to provide context specific solutions. However, several studies found that decentralisation does not always enhance the desired outcomes [63-65]. We noticed that, in all three study countries, weak leadership and limited management and strategic thinking skills at decentralised level hamper the implementation of strategies needed to increase access to quality newborn care for the most vulnerable.

### Limitations of this study

The study has several important limitations. First, because this study was a short term consultancy assignment time and resource constraints made it impossible to conduct an in-depth analysis of the complexities of newborn care. However, we tried to be as comprehensive as possible by covering all health system building blocks and their specificities for newborn care [11]. Secondly, due to the study set-up, audio-recording and full transcription of interviews was not possible. Third, because of time constraints the field visits included only a few districts and health facilities in each study country. The newborn healthcare situation might be different in other districts. Fourth, because participation at interviews was voluntary, it might have resulted in selection bias. Fifth, because only available data on newborn care was used for the situation analysis, not all aspects of newborn care could be assessed thoroughly by lack of data.

## Conclusion

In Indonesia, Lao PDR and the Philippines we identified the need and opportunity to improve access to good quality newborn care. There is an urgent need to address weak leadership and governance regarding newborn care, the quality of newborn care provided and inequities in newborn care. Only then can newborn mortality and morbidity decrease in these three countries. Child survival programmes focussed on children over four weeks of age have shown to have positive outcomes. Similar efforts as those used in these programmes should be considered in newborn care.

## Competing interests

Dr Nabila Zaka and Mr Basil Rodriques are employees of ‘UNICEF East Asia and Pacific Regional Office’ who financed this study.

## Authors’ contributions

*Els Duysburgh* was the overall study coordinator and end responsible and developed together with Birgit Kerstens the study design and study tools. She coordinated the newborn care needs assessment in Indonesia and as such participated in data collection (document review, key informant interviews and health facility visits), data interpretation and report writing in Indonesia. She coordinated the writing of this paper. *Birgit Kerstens* developed together with Els Duysburgh the study design and study tools. She coordinated the newborn care needs assessment in Lao PDR and as such participated in data collection (document review, key informant interviews and health facility visits), data interpretation and report writing in Lao PDR. She contributed to the writing of this paper by giving inputs on the general context of the paper. *Melissa Diaz* coordinated the newborn care assessment in the Philippines and as such participated in data collection (document review, key informant interviews and health facility visits), data interpretation and report writing in the Philippines. She contributed to the writing of this paper by giving inputs on the general context of the paper. *Vini Fardhdiani* participated in data collection (document review, key informant interviews and health facility visits), data interpretation and report writing in Indonesia. She contributed to the writing of this paper by giving inputs and checking statements regarding newborn care in Indonesia. *Katherine Ann V. Reyes* participated in data collection (document review, key informant interviews and health facility visits), data interpretation and report writing in the Philippines. She contributed to the writing of this paper by giving inputs and checking statements regarding newborn care in the Philippines. *Khamphong Phommachanh* participated in data collection (document review, key informant interviews and health facility visits), data interpretation and report writing in Lao PDR. She contributed to the writing of this paper by giving inputs and checking statements regarding newborn care in Lao PDR. *Marleen Temmerman* gave inputs to and supported the paper writing and reviewed the final draft. *Basil Rodriques* participated in the selection of the study countries. He gave inputs to the different country reports written as part of the study and he gave inputs to this paper. *Nabila Zaka* conceptualized and supervised the study for UNICEF East Asia and Pacific Regional Office. She coordinated the initiation and implementation of the study with UNICEF country focal points and facilitated the government approval process in the study countries. She gave inputs to the study design and study tools. She gave comments and inputs on data interpretation and gave inputs to the country reports written as part of the study. She contributed to the writing of this paper by giving inputs on the general context of the paper. All authors read and approved the final manuscript.

## Pre-publication history

The pre-publication history for this paper can be accessed here:

http://www.biomedcentral.com/1471-2431/14/46/prepub
